# Microfluidic platform for nanoliter qPCR of several retinal pigment epithelial (RPE) cells

**DOI:** 10.1088/1361-6439/ae9c4b

**Published:** 2026-09-01

**Authors:** Debdyuti Mandal, Akash Roy, Xuelian Chen, Jiang F Zhong, Eun Sok Kim

**Affiliations:** 1 Department of Electrical and Computer Engineering, University of Southern California, Los Angeles, CA 90089, United States of America; 2 Keck School of Medicine, University of Southern California, Los Angeles, CA 90033, United States of America; 3 Department of Basic Sciences, School of Medicine, Loma Linda University, Loma Linda, CA 92350, United States of America

**Keywords:** droplet microfluidics, automated quality control, retinal pigment epithelium, single-cell qPCR, electrocoalescence

## Abstract

The clinical implant of stem cell–derived retinal pigment epithelium (RPE) monolayer for age-related macular degeneration requires rigorous validation of the monolayer’s cellular maturity. Conventional bulk molecular assays lack the sensitivity to resolve cellular heterogeneity and are prone to damaging the RPE monolayer. This study developed and validated an automated nanoliter-scale microfluidic platform for single-cell transcriptomic quality control of engineered RPE monolayers. A compact polydimethylsiloxane -based microfluidic platform was developed to generate monodisperse 10 nL RPE cell cDNA droplets and 90 nL quantitative PCR (qPCR) reagent droplets using dual-focused-flow geometries. Deterministic droplet fusion was achieved via direct current electrocoalescence, enabling ∼100% merging efficiency. An auxiliary co-flow spacing mechanism was implemented to prevent secondary coalescence during downstream transport. Automated droplet collection into oil-filled 96-well plates was achieved through a custom two-dimensional gantry system, and droplet volume consistency was verified using a Python-based computer vision algorithm. Biological validation was conducted using H14 human embryonic stem cell–derived RPE cells targeting *β*-actin and lineage-specific markers MITF1, MITF2, PEDF, and PMEL17. The platform demonstrated stable generation and deterministic merging of nanoliter droplets, yielding uniform 100 nL reaction volumes suitable for qPCR analysis. Automated volumetric verification confirmed high droplet uniformity and reliability. Gene expression analysis revealed robust detection of housekeeping and lineage-specific genes at nanoliter scales. These findings demonstrated the feasibility of performing nanoliter-scale qPCR using the proposed microfluidic workflow. The workflow enabled reliable detection of extremely low quantities of nucleic acid using a widely available commercial qPCR platform, providing a practical and accessible approach for routine laboratory and translational research applications. The system-maintained assay sensitivity while significantly reducing reagent consumption and sample input. This automated microfluidic platform enabled reproducible, sample-efficient, single-cell transcriptomic validation of stem cell–derived RPE monolayers. By integrating droplet generation, deterministic merging, automated collection, and computational verification, the system addressed key limitations of bulk molecular quality control of RPE monolayers. This work established a scalable and cost-effective nanoliter qPCR framework for high-resolution molecular quality assurance of regenerative cell therapies.

## 1. Introduction

Age-related macular degeneration (AMD) is a leading cause of irreversible vision loss and an age-onset form of retinal degeneration, affecting more than 200 million individuals worldwide, predominantly individuals over 50 years of age [1–4]. A hallmark of AMD progression is degeneration of the retinal pigment epithelium (RPE), which ultimately drives disease advancement into dry AMD with geographic atrophy (85%–90% of cases) or wet AMD characterized by choroidal neovascularization (10%–15%) [1, 5]. Despite extensive clinical efforts, there is currently no curative therapy for AMD, and existing treatments are limited mainly to slowing disease progression. As a result, both pharmacologic and cell-based therapeutic strategies are actively being explored, with numerous candidates currently under clinical evaluation. While pharmacologic interventions—including anti-angiogenic, complement-inhibitory, anti-amyloid, and antioxidant therapies—have shown efficacy in delaying vision loss, particularly in wet AMD, their effectiveness diminishes once RPE degeneration progresses to advanced disease stages [1]. In this context, transplantation of RPE monolayers has emerged as a promising cell-based strategy to preserve or restore retinal function by supporting photoreceptor survival [6–11]. However, the clinical translation of RPE-based therapies places stringent demands on the manufacturing, characterization, and quality control of RPE implants. In particular, RPE monolayers derived from induced pluripotent stem cells (iPSCs) must be validated as functionally mature and spatially uniform tissues, with appropriate ultrastructural organization, transcriptional identity, and maturation state [12–14]. Failure to adequately characterize these properties can result in the presence of damaged or dedifferentiated cells, which may trigger TGF*β*-mediated epithelial-to-mesenchymal transition (EMT), loss of RPE-specific gene expression, and cellular senescence, ultimately compromising therapeutic efficacy [9]. From an engineering perspective, these challenges underscore a critical unmet need for scalable, high-resolution molecular profiling tools capable of assessing RPE quality at the level of individual cells or defined microdomains while preserving tissue integrity. Conventional bulk assays lack the spatial resolution and sensitivity required to detect subtle heterogeneity within RPE monolayers. In contrast, existing analytical workflows are often labor-intensive, sample-intensive, and poorly suited for integration into good manufacturing practice (GMP) pipelines.

Single-cell quantitative PCR (qPCR) on microfluidic platforms requires precise, deterministic control over droplet-based reaction handling, particularly for the timed merging of reagent-containing droplets with cell-laden compartments at nanoliter volumes. Variability in droplet synchronization, flow stability, or electrohydrodynamic actuation can directly compromise amplification efficiency, transcript recovery, and quantitative accuracy—posing a significant barrier to scalable single-cell gene expression analysis. To address these challenges, we present a compact and cost-effective polydimethylsiloxane (PDMS) microfluidic platform that enables highly reproducible nanoliter droplet merging via direct current (DC) electrocoalescence, specifically designed to support integrated single-cell qPCR workflows.

Conventional qPCR assays are typically performed in microliter-scale reaction volumes (≈10 *µ*l) using manual pipetting and multi-well plate formats [15]. While widely adopted, this approach presents inherent limitations when applied to low-input samples, rare-cell populations, or single-cell analyses [16]. Large reaction volumes dilute nucleic acid templates, reduce effective local concentrations, and increase reagent consumption, thereby elevating cost and potentially compromising sensitivity. Furthermore, manual pipetting can introduce variability in reagent dispensing and mixing, resulting in increased inter-assay variability, particularly when handling small sample volumes [17]. Open handling steps also increase the risk of contamination and sample loss, challenges that become increasingly significant in workflows requiring high precision and molecular fidelity. Microfluidics-based qPCR platforms address these limitations by enabling deterministic handling of nanoliter- to picoliter-scale reaction volumes within closed, highly controlled environments. This solves the problem of variability when the reagent volume is low, since we can perform qPCR on the same reagent spread across multiple wells [18, 19]. At reduced length scales, diffusion distances decrease substantially, leading to faster mixing kinetics and improved reaction uniformity without reliance on turbulent convection. Smaller volumes effectively increase the template-to-reagent ratio, thereby enhancing amplification efficiency and sensitivity, particularly for single-cell or ultra-low-copy-number targets [20]. In addition, precise microfluidic control over droplet generation, pairing, and merging enables automated, reproducible reagent metering, minimizing operator-induced variability [21]. The closed microfluidic architecture further reduces contamination risk and allows seamless integration with upstream processes such as single-cell isolation, extracellular vesicle enrichment, or on-chip lysis [22]. Beyond analytical improvements, microfluidic qPCR significantly reduces reagent consumption, lowering per-assay costs and enabling scalable high-throughput operation. Thermal cycling can also be more efficient due to improved heat transfer characteristics at small volumes, potentially shortening amplification times and improving reaction consistency [23]. Collectively, these advantages position microfluidics-based qPCR platforms as a powerful alternative to conventional pipette-based methods, particularly for applications requiring high sensitivity, minimal sample input, enhanced reproducibility, and integration into automated, next-generation molecular diagnostic workflows. However, translating nanoliter-scale qPCR into robust and scalable workflows requires not only confined reaction chambers but also reliable strategies for controlled reagent delivery and reaction initiation [15].

While commercial droplet digital PCR (ddPCR) platforms have become widely accessible standard tools for nucleic acid quantification, they operate as closed, rigid ecosystems that lack the functional flexibility required for integrated, end-to-end cell therapy pipelines. Standard commercial systems are fundamentally designed for simple, bulk-emulsified ‘single-pot’ reactions; they template a pre-mixed solution into stochastic droplets for end-point fluorescence readings. This architecture cannot accommodate dynamic, multi-reagent synchronization, such as the active pairing and timed merging of a rare cellular lysate droplet with a separate upstream reverse transcription or master mix droplet. Furthermore, standard ddPCR systems lack built-in mechanism options to programmatically extract, sort, and deposit individual nanoliter reaction volumes onto open multi-well substrates (e.g. 96-well plates) for custom downstream profiling or integration with automated GMP robotic gantries.

Beyond these fundamental functional limitations, commercial ddPCR instruments and their proprietary, consumable microfluidic cartridges present a high financial barrier to entry, often requiring capital equipment investments exceeding $100 000. To resolve both the technical restrictions and the cost constraints of commercial alternatives, we engineered a custom, open-architecture platform using cost-effective fabrication techniques. By utilizing benchtop 3D-printed molds for device fabrication and off-the-shelf automation components (such as an Arduino microcontroller and standard stepper motors), the total component cost of our complete dual-generation, electrocoalescence, and 2D gantry system is estimated to be less than $1500. This custom framework not only introduces the missing capability of deterministic, multi-stream nanoliter reagent merging and automated fraction collection but also demonstrates that high-resolution, single-cell quality control can be achieved at a fraction of the capital cost of commercial alternatives.

**Figure 1.**
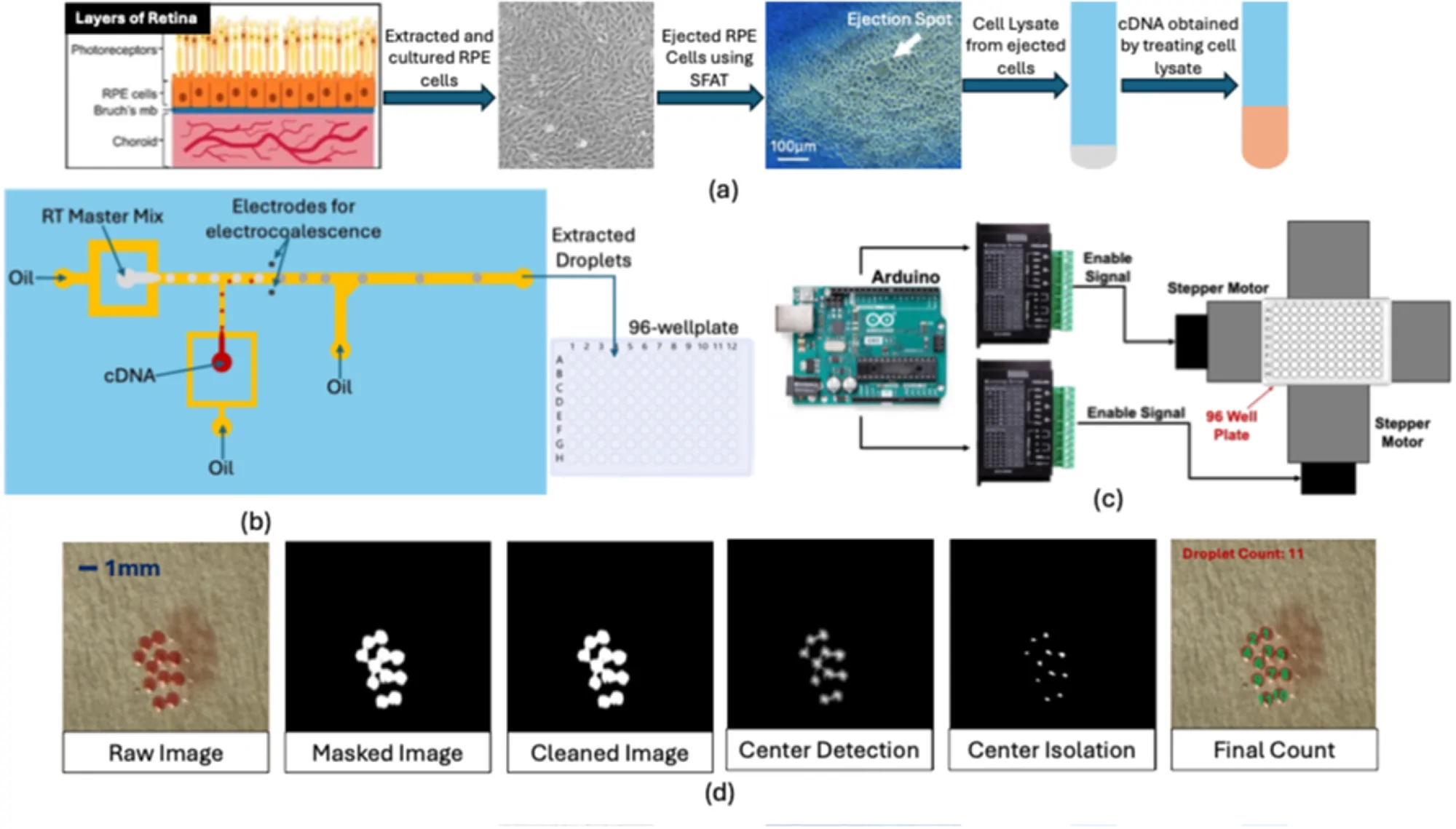
(a) Extraction of cDNA from Retinal Pigmented Epithelial (RPE) cells extracted from a cultured RPE monolayer after a few cells are ejected from the monolayer by an acoustic droplet ejector [24], (b) Overview of the microfluidic channel for droplet merging and spacing. (c) An automated collection system of droplets into a 96-well plate. (d) Computer vision-based droplet identification for volumetric control.

In this work, the system (illustrated in figure 1) employs dual focused-flow geometries to independently generate monodisperse oil-encapsulated aqueous droplets of 10 nL and 90 nL, corresponding to RPE cell cDNA and reagent-containing compartments, respectively. Stable droplet generation and inter-droplet spacing are achieved through peristaltic pumping combined with pulse dampers, ensuring consistent synchronization prior to merging. Two distinct droplet streams are guided to a T-junction, where electrocoalescence is initiated using needle electrodes. This work introduces a microfluidic platform that utilizes DC electrocoalescence to achieve highly efficient droplet merging for qPCR applications. In addition, the study details the optimization of electrode geometry, applied voltage, and flow parameters to ensure stable operation while maintaining droplet integrity and preserving reagent functionality. The DC electrocoalescence enables deterministic, 100% merging without disrupting upstream droplet formation. An orthogonal electrode configuration further enhances robustness, with the coalescence location governed by inter-droplet spacing rather than stochastic field effects. This controlled and scalable droplet-merging strategy provides a reliable foundation for nanoliter-scale reverse transcription and PCR amplification, offering a practical route toward high-throughput, low-volume single-cell gene expression analysis on lab-on-a-chip platforms. Although droplet generation has been reported in the literature, precise extraction of the formed droplets with reasonable control over the number of droplets has not been established in detail. Using an additional co-flow to the channel after droplet coalescence, we increase the inter-droplet spacing. By varying the co-flow rate, we achieve a stable inter-droplet spacing, preventing droplets from self-coalescing within the collection tubing. The droplets are automatically collected in an oil-filled 96-well plate using a 2D gantry system controlled by an Arduino. The collected droplets are counted using a computer vision model to confirm the reagent volume, and are then sent for gene expression analysis. We have assessed the expression of representative housekeeping and key pluripotency genes for evaluating transcriptional integrity and cellular state. Unlike conventional digital droplet PCR, which relies on stochastic partitioning and endpoint quantification, the present work introduces a hybrid droplet-microfluidic qPCR workflow that combines deterministic nanoliter droplet handling, DC electrocoalescence, automated collection, and real-time qPCR for low-input transcriptomic analysis.

## 2. Materials and methods

### 2.1. Microfluidic device fabrication

**Figure 2.**
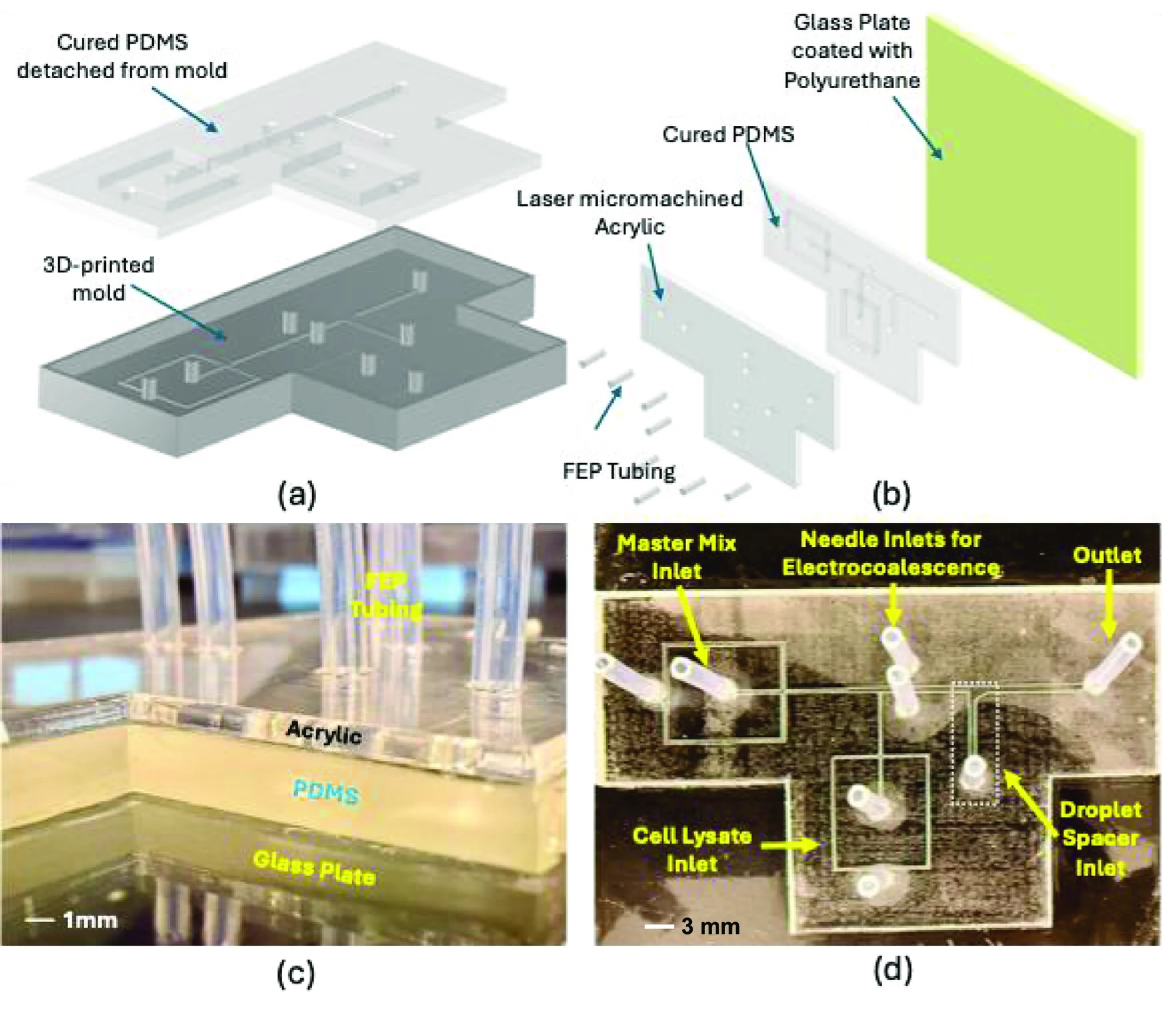
(a) Illustration of the 3D-printed mold and PDMS layer peeled from the mold. (b) Illustration of how the microchannel is enclosed, and connected to the tubing. (c) and (d) Perspective and top-view photos of the fabricated microfluidic platform, respectively.

The PDMS microfluidic device was fabricated using a stereolithography (SLA)-based 3D-printed mold (Formlabs Form 3B+, Gray Resin V4), in which the microchannel features were designed as positive protrusions with integrated inlet and outlet ports for direct FEP tubing insertion (figure 2). Following resin cleaning and UV post-curing, the mold was surface treated to facilitate reliable PDMS curing and de-molding. PDMS (Sylgard 182, 10:1 base-to-curing agent ratio) was cast onto the mold, degassed, thermally cured, and subsequently peeled from the mold. The PDMS replica was permanently bonded to a polyurethane-coated glass substrate and reinforced with a laser-machined acrylic cover plate to improve structural rigidity during tubing integration. Finally, FEP tubing was inserted into the integrated ports and sealed using UV-curable resin to provide robust, leak-free fluidic connections. A detailed description of the fabrication procedure is provided in the supporting information.

### 2.2. Experimental setup for the microfluidic system

**Figure 3.**
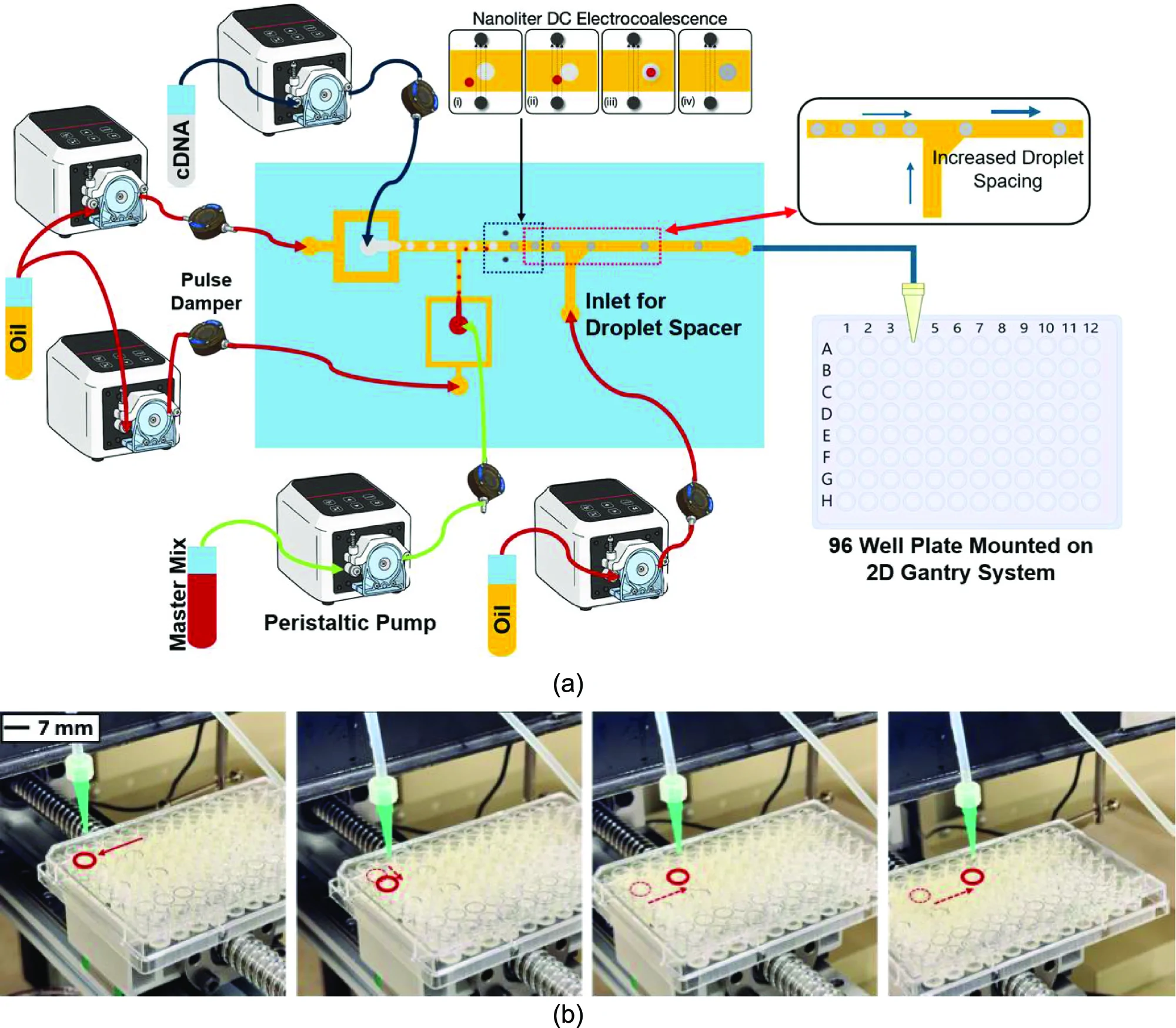
(a) Schematic of the fluidic connections for a microfluidic platform using a peristaltic pump and a damper for each inlet, showing DC electrocoalescence and droplet spacing and final collection in a well plate. (b) Sequential photos showing the stage movement with a single droplet being dropped into each individual well for automated droplet collection using a 2-axis stage controlled by an Arduino (figure S1).

The microfluidic platform comprises two independent flow-focusing droplet generators with channel widths of 554 *µ*m and 266 *µ*m, designed to produce monodisperse aqueous droplets with volumes of 90 nL and 10 nL, respectively, operating within the transitional flow regime (figure 3(a)). These two droplet streams converge at a downstream T-junction, where electrodes are integrated to enable electrically induced droplet coalescence. To systematically investigate electrocoalescence behavior, orthogonal electrode geometry (orthogonal to the main channel) is evaluated. Electrical connections are established using 3 M NaCl-filled tubing as liquid electrodes, coupled to syringe-needle interfaces, and energized with DC voltage. The device incorporates four inlet channels, each independently controlled by peristaltic pumps, with pulse dampers to minimize flow fluctuations and ensure stable, repeatable droplet generation. This configuration enables precise control over droplet size, spacing, and synchronization before merging. Under optimized DC electrocoalescence conditions, reliable and deterministic merging of 10 nl and 90 nl droplets is achieved, resulting in uniform 100 nL merged droplets without perturbing upstream droplet formation.

### 2.3. Droplet collection system

To facilitate automated sample recovery, the microfluidic outlet is integrated with a flexible silicone tube terminated with a tapered tip, which optimizes pinch-off dynamics to ensure a consistent number of aqueous droplets per oil segment. To optimize the dispensing process and eliminate droplet coalescence, experiments were conducted using multiple tapered dispensing tips with inner diameters ranging from 0.25 mm to 2.0 mm. Surface tension was identified as a critical parameter governing droplet detachment and stability. By optimizing the dispensing conditions, coalescence of aqueous droplet was prevented within the continuous oil phase during droplet generation. Additionally, the process was tuned to ensure that each oil droplet consistently encapsulated an identical number of aqueous droplets, thereby improving the uniformity and reproducibility of the generated emulsions. The performance of each dispensing tip was systematically evaluated based on the achievable flow rates and the extent of droplet coalescence. Among the tested configurations, the 0.5 mm tapered tip provided the most stable operation, yielding the highest consistency in droplet formation while effectively preventing coalescence. This assembly was interfaced with a custom-engineered 2D gantry system mounted on a rigid frame and driven by high-resolution stepper motors (figure S1 in supplemental information). Controlled by an Arduino Uno microcontroller and powered by a 24 V DC supply, the stage is programmed to translate with spatial precision matching the standard 9 mm center-to-center pitch of a 96-well plate (figure 3(b)).

The fractionation process is governed by a programmed duty cycle in which the gantry maintains a 1 s dwell at each well coordinate to ensure the complete transfer of the falling droplet before advancing to the next position. To preserve the structural integrity of the encapsulated phases, each receiving well is preloaded with 50 *µ*l of carrier oil. This oil layer serves as a kinetic buffer, effectively dampening impact forces and preventing splashing or coalescence that typically occur during high-velocity droplet collection. This automated sequence is repeated across the entire plate until all target rows are populated, ensuring high-speed sample collection for downstream analytical applications.

### 2.4. Droplet reagents for the detection of gene expression in RPE cells

H14 human embryonic stem cell-derived retinal pigment epithelial (RPE) cells were cultured in X–VIVO™ 10 medium supplemented with 10 *µ*M ROCK inhibitor Y-27 632 until a confluent monolayer exhibiting uniform morphology was established. Cells were subsequently isolated using an acoustic droplet ejector as previously described [24]. Prior to ejection, cells were fluorescently labeled to enable visualization and viability assessment using membrane, nuclear, and dead-cell stains. Individual acoustically ejected cells were collected for whole-transcriptome amplification, followed by real-time qPCR to evaluate the integrity of the amplified cDNA and confirm the expression of housekeeping (*β*-actin) and RPE-specific marker genes (MITF1, MITF2, PEDF, and PMEL17). For each target gene, qPCR was performed on three independent plates (designated Plate 1, Plate 2, and Plate 3), and the corresponding plate-level *C*_t_ values are reported in the Results section. Detailed cell culture conditions, fluorescent labeling procedures, whole-transcriptome amplification protocol, qPCR conditions, and primer sequences are provided in the Supporting Information.

## 3. Results and discussion

### 3.1. Precise droplet production and alignment

In microfluidic systems, fluid flow is governed by a combination of inertial, viscous, gravitational, and capillary forces [25]. Among these, the capillary number (*Ca*) is one of the most significant and widely used dimensionless parameters for characterizing droplet generation [21]. It is defined as the ratio of viscous shear forces to capillary pressure, providing a quantitative measure of the relative influence of viscous effects over interfacial tension. Additionally, the dimensionless Reynolds number (*Re*), relatively low in microfluidic systems, plays a key role in characterizing inertial effects and flow stability. The capillary and Reynolds numbers can be obtained with

\begin{equation*}{C_a}\, = \,\frac{{\mu u}}{\sigma }\end{equation*}

\begin{equation*}{\operatorname{R} _e}\, = \,\frac{{\rho uD}}{\mu }\end{equation*}

where *μ* is the dynamic viscosity of the dispersed phase (Pa-s); *u* is the velocity of the dispersed phase (m/s); *σ* is the interfacial surface tension (N m); and *D* is the fluidic hydraulic diameter (m). At higher *Ca*, viscous forces dominate, leading to smaller and more frequent droplets, while the microfluidic system may fail to pinch off the dispersive phase, resulting in a long tail of dispersive phase within the channel and, consequently, either improper or no droplet generation. On the other hand, at lower *Ca*, surface tension governs, resulting in larger and more frequent pinching-off events leading to stable droplet formation. In the transitional regime, moderate *Re* contributes to controlled droplet pinch-off and uniform spacing, ensuring reproducible droplet generation, which is critical for downstream applications. The flow regimes in droplet microfluidics are highly dependent on the phase flow rate ratio *ϕ*,

\begin{equation*}\varphi {\mkern 1mu} = {\mkern 1mu} \frac{{{\mathrm{Flow}}{\mkern 1mu} \,\,\,{\mathrm{Rate}}{\mkern 1mu} \,\,\,{\mathrm{of}}{\mkern 1mu} \,\,\,{\mathrm{Dispersive}}{\mkern 1mu} \,\,\,{\mathrm{Phase}}}}{{{\mathrm{Flow}}{\mkern 1mu} \,\,\,{\mathrm{Rate}}{\mkern 1mu} \,\,\,{\mathrm{of}}{\mkern 1mu} \,\,\,{\mathrm{Continuous}}{\mkern 1mu} \,\,\,{\mathrm{Phase}}}}.\end{equation*}

**Figure 4.**
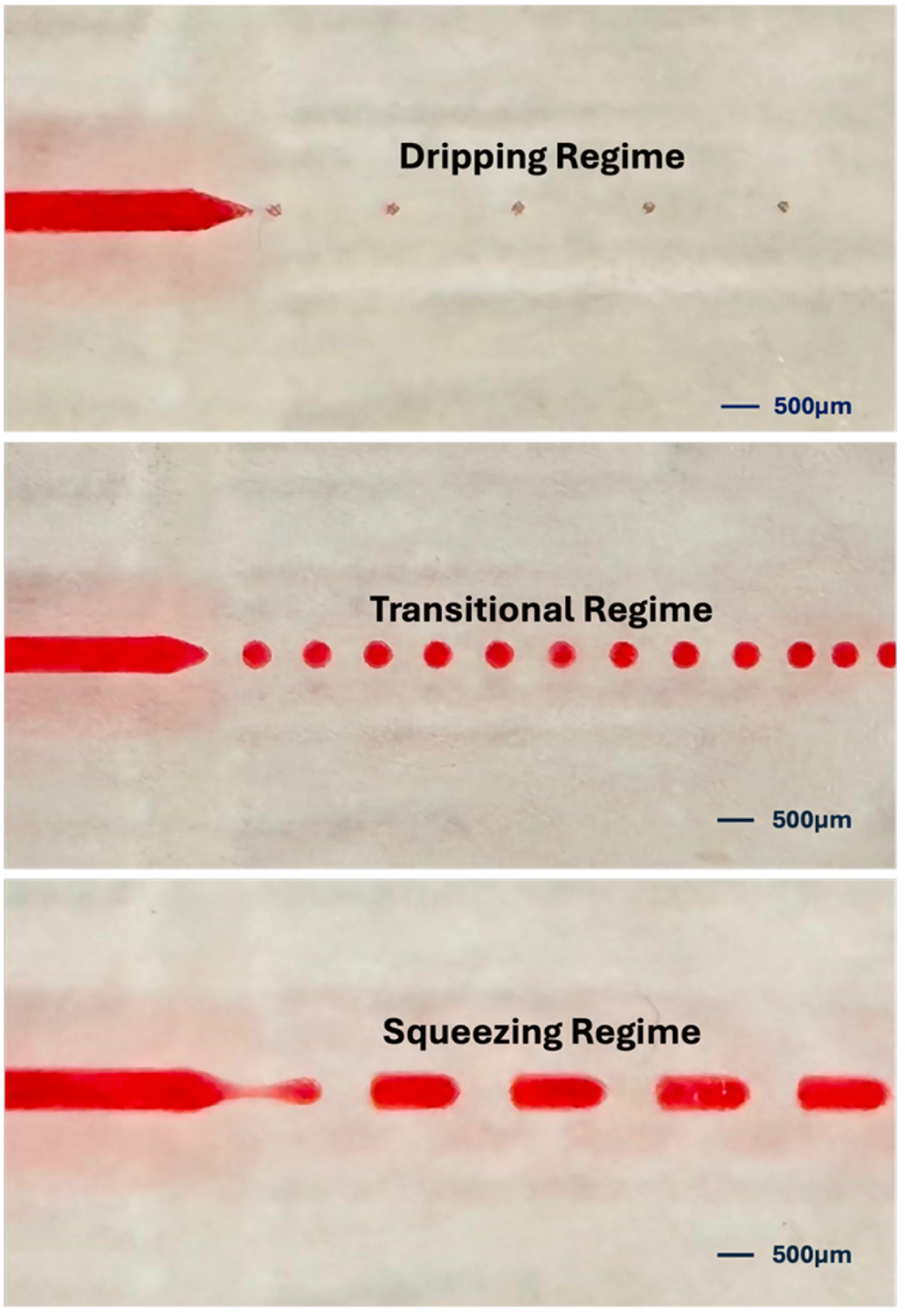
Different regimes of droplet formation by varying the phase flow rate ratio between the continuous and the dispersive media.

As the microfluidic system operates under a focused flow condition, we have investigated different flow regimes by varying the phase flow rate ratio (*ϕ*) under a constant dispersed-phase flow rate (figure 4). For our channel dimensions, if *ϕ* < 0.3 (obtained by adjusting the flow rates of the oil and the aqueous solution), the droplet generation was in the dripping regime, where the droplet diameter is less than the channel width and is not well controlled. For 0.4 < *ϕ* < 0.63 (which falls into the transitional regime), the droplets are spherical, with a diameter about equal to the channel width, which is ideal for our application. For 0.7 < *ϕ* < 1, the droplets are generated in a squeezing regime and are shaped elliptically, with their volumes poorly controlled.

The microfluidic platform employs 554 *μ*m and 266 *μ*m channels to generate ∼90 nl and ∼10 nl droplets of RT master mix and cDNA analogs, respectively, via a flow-focusing configuration operating in the transitional flow regime. Under optimized flow conditions, both channels produce monodisperse spherical droplets. Deterministic droplet pairing and controlled coalescence at a T-junction are achieved by independently tuning four peristaltic pumps to regulate the continuous and dispersed phases. To evaluate droplet monodispersity, the generated droplets were quantitatively characterized under the optimized operating conditions. Individual frames were extracted from video recordings of droplet generation within the transitional regime, and the diameters of approximately 100 droplets were measured using a calibrated pixel-based image analysis method. With the known microchannel width serving as the spatial reference, the droplet monodispersity was quantified by calculating the coefficient of variation (CV) from the measured droplet diameters. A CV of 3.6% was obtained, indicating a narrow droplet size distribution and confirming highly monodisperse droplet generation. The same operating conditions and input parameters were maintained throughout all experiments to ensure consistent and reproducible droplet formation. Temporal synchronization of droplet arrival was maintained through iterative pump adjustments, as minor variations in flow rates significantly affect droplet timing, spacing, and pairing. Stable flow conditions preserve consistent droplet formation frequency and spacing, enabling near one-to-one alignment at the junction. This precise coordination minimizes stochastic effects that typically reduce coalescence efficiency in multi-inlet systems, resulting in reproducible droplet collisions and reliable merging, demonstrating robust control over nanoliter-scale, multi-reagent droplet handling. Experimental results indicate that this active flow control strategy achieves an alignment accuracy of ∼94%, confirming that nearly all droplet pairs meet predictably in both space and time. These findings highlight the critical role of dynamic flow balancing in droplet microfluidics, where small fluctuations in flow rate or channel backpressure can substantially shift droplet timing. By effectively decoupling droplet generation from merging dynamics, the platform provides precise control over droplet trajectory, frequency, and spacing before downstream electrocoalescence. This deterministic control enhances droplet pairing efficiency and ensures reproducible performance across repeated trials, establishing a foundation for reliable on-chip reagent mixing and single-cell qPCR workflows.

### 3.2. DC electrocoalescence

**Figure 5.**
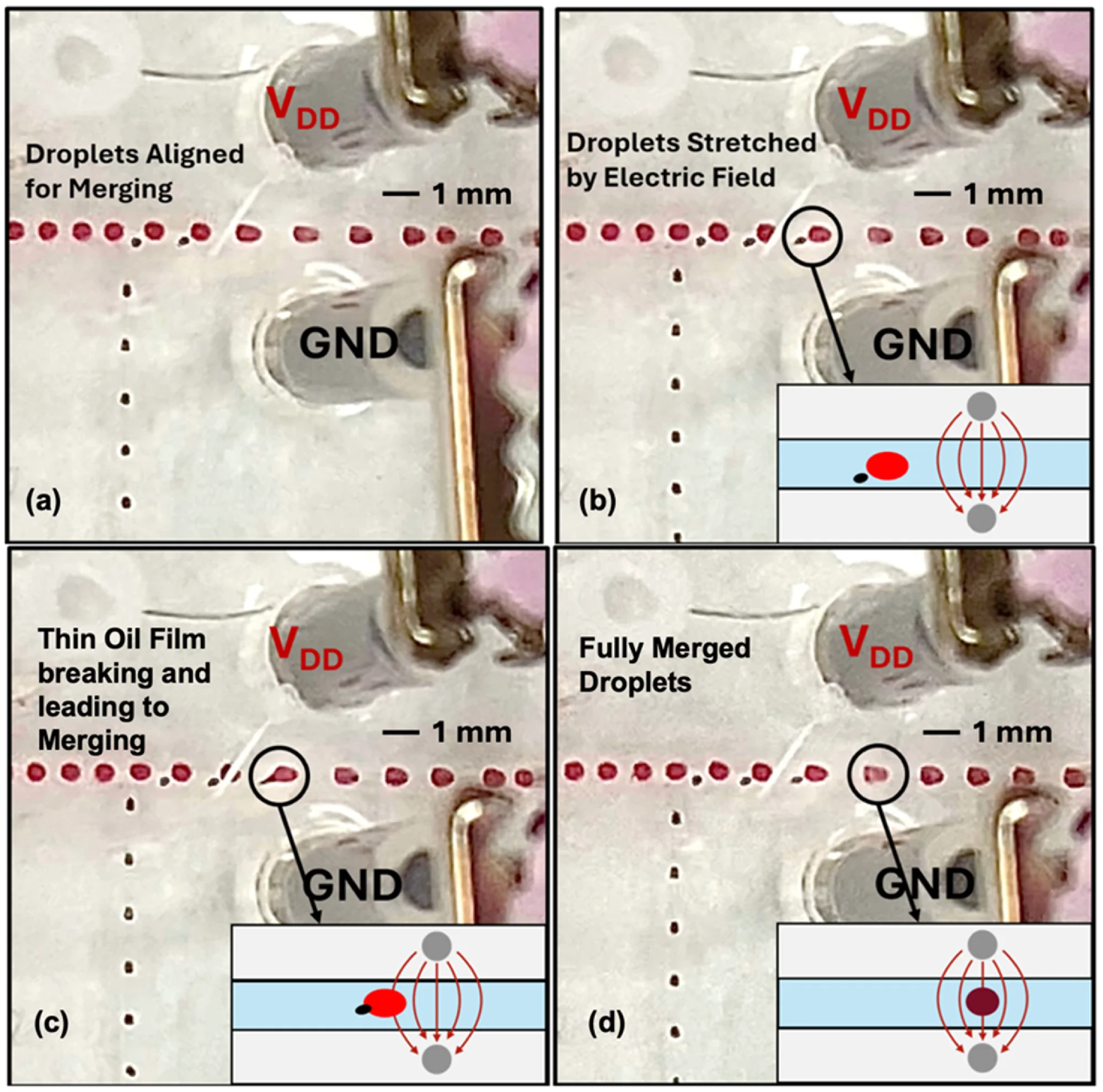
Sequential photos showing precise alignment and electrocoalescence of 90 nL of red droplet and 10 nL of black droplet. (a) The two droplets are precisely aligned at the junction. (b) The droplets are stretched due to the presence of the DC electric field. (c) The thin oil film between the two droplets breaks leading to the merging. (d) The droplets are finally merged resulting into single uniformly sized droplet with a combined volume of 100 nl.

To achieve precise mixing between the 90 nl RT master mix droplet and the 10 nl RPE cDNA droplet, an electrocoalescence was used. In contrast to conventional AC-field actuation, a DC electric field substantially enhances electrocoalescence efficiency and operational stability. The DC electric field was established by biasing the integrated microelectrodes using a regulated DC power supply, with the output voltage subsequently increased via a portable DC–DC boost converter to achieve the required electric field (figure 5). This configuration enables precise and continuous control of the electric field amplitude across the droplet interaction region. The DC electrocoalescence process is governed by the interaction of the applied electric field with adjacent aqueous droplets suspended in the continuous phase. Upon application of a DC electric field, the conductivity and permittivity mismatch between the aqueous and oil phases induces interfacial polarization, resulting in the accumulation of opposite charges at the droplet interfaces. The resulting electrostatic attraction drives adjacent droplets toward one another, while the electric field simultaneously promotes drainage and progressive thinning of the intervening oil film. As the oil layer reaches a critical thickness, intermolecular attractive forces overcome the stabilizing effects of the interface, leading to film rupture and the formation of a liquid bridge between the droplets. This bridge rapidly expands under the action of surface tension, ultimately resulting in complete droplet coalescence. The efficiency of this process is governed by the applied electric field, droplet spacing, fluid properties, and interfacial tension, all of which influence the rate of film thinning and the onset of coalescence. Droplet merging begins at an applied voltage of approximately 250 Vpp, which corresponds to the minimum electric field required to generate sufficient electrohydrodynamic stresses and interfacial polarization for droplet–droplet interaction. At this threshold, however, coalescence events are intermittent, and the overall merging efficiency remains relatively low, suggesting that the induced electric stresses are insufficient to consistently overcome stabilizing interfacial forces. Importantly, across this voltage regime, the droplet generation process, transport dynamics, and arrival frequency at the junction remain highly stable and unaffected by the applied DC field. This observation confirms that the electric actuation is effectively localized to the coalescence region and does not perturb upstream hydrodynamic droplet formation.

Preserving stable droplet timing and spacing under DC excitation enables droplets to converge at the junction with highly repeatable spatial alignment and temporal synchronization. Consequently, the DC field affords superior spatial and temporal resolution during droplet pairing and interaction, facilitating more predictable coalescence behavior. Through systematic voltage optimization, a monotonic improvement in coalescence efficiency is observed with increasing DC field. At an applied voltage of 550 Vpp, the electric field consistently destabilizes the thin oil film separating adjacent droplets, leading to rapid interfacial rupture and fully deterministic merging. Under these optimized conditions, 100% coalescence efficiency was achieved for all paired droplet events, with no observable missed merging events across repeated trials. Each coalescence event produces a single, uniformly sized droplet with a combined volume of approximately 100 nl. These results demonstrate that DC-driven electrocoalescence provides a robust, highly controllable mechanism for droplet merging in microfluidic systems. By decoupling hydrodynamic droplet generation from electrically induced merging dynamics, this approach enables precise control over droplet interaction timing, location, and outcome. The observed stability and repeatability across voltage conditions highlight the suitability of DC electrocoalescence for complex, multi-step microfluidic workflows, particularly those requiring reliable reagent mixing and volume-defined droplet fusion. Although peristaltic pumps inherently generate pulsatile flow, the incorporation of pulse dampers effectively minimized flow fluctuations, resulting in stable and reproducible droplet generation throughout the experiments. The damped flow produced a highly consistent interdroplet spacing, which was critical for maintaining a constant droplet generation frequency. This consistency was particularly important because any variation in the interdroplet distance could alter the arrival times of the 90 nL and 10 nL droplets at the cross-junction, thereby disrupting their synchronization and adversely affecting the overall operation of the system. While a small degree of residual pulsation remained, its impact on device performance was minimal, as demonstrated by an average droplet alignment efficiency of approximately 94%, indicating reliable long-term synchronization and stable device operation. Figure 5 illustrates the precise alignment of 90 nL RT master mix droplets (red dye for indication) and 10 nL cDNA droplets (black dye for indication) with 94% accuracy and nearly 100% merging of the two droplets.

### 3.3. Controlling droplet spacing for prevention of droplet self-merging without surfactant

While surfactants are commonly used in droplet microfluidics to prevent droplet merging, their presence is counterproductive in systems requiring active, voltage-induced merging. Surfactants adsorb at the oil-water interface, creating steric or electrostatic barriers that significantly reduce interfacial tension and inhibit the film drainage required for coalescence. Such a stabilization technique would require higher voltage and could compromise the efficiency of the DC electrocoalescence. If the electrocoalesced droplets (consisting of 90 nl of qPCR master mix and 10 nl of cDNA) transition from the microfluidic channel into a larger-diameter outlet tube, the increased cross-sectional area of the outlet tube lowers the flow velocity and, thus, the distance between consecutive droplets. This effect is further exacerbated in the vertical segments of the tubing, where the mass density difference causes different buoyancy forces on the oil and the aqueous solution, making the droplets non-spherical and leading to unintentional merging. To counteract this, an auxiliary oil inlet was positioned downstream of the DC coalescence zone, to act as a droplet spacer. The droplet spacer increases the inter-droplet spacing by augmenting the total volumetric flow rate within the microchannel without altering droplet production frequency. For a cross-sectional area of the microfluidic channel *A* and the droplet generation rate *f*, the final center-to-center droplet distance with the droplet spacer is given by

**Figure 6.**
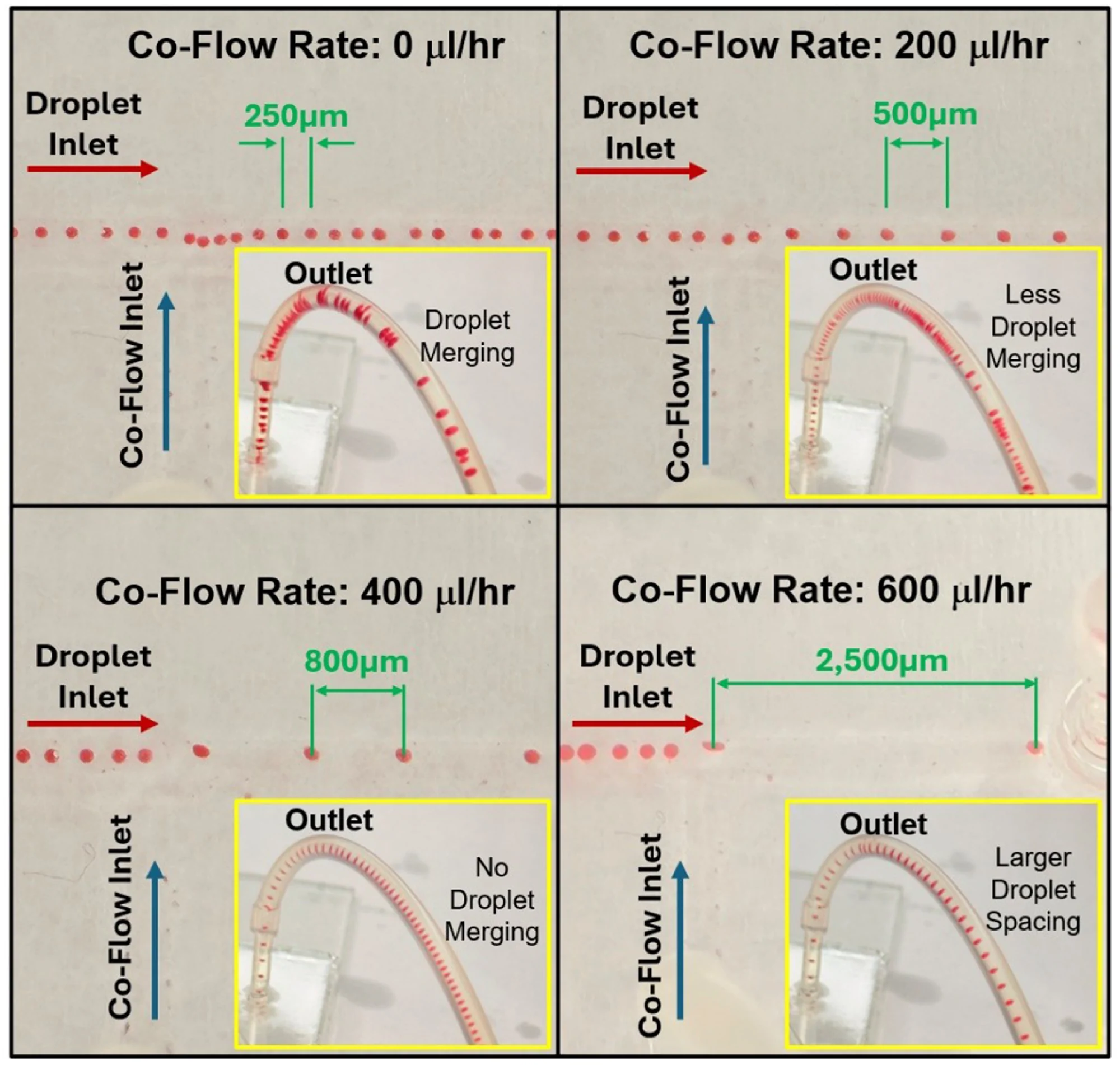
Photos showing inter-droplet spacing along with droplet condition at the output tubing by varying the flow rate of the co-flow inlet.

\begin{equation*}{L_s}\, = \,\frac{{{Q_c}\, + {Q_d} + {Q_s}}}{{fA}}\end{equation*}

where *Q*_c_, *Q*_d_, and *Q*_s_ are the flow rates of the continuous, dispersive, and spacer systems, respectively. By introducing a flow (*Q*_s_) at a rate significantly higher than the flows (*Q*_c_ and *Q*_d_) in the primary channel while maintaining the same channel dimensions, the local velocity substantially increases, leading to larger inter-droplet distance that prevents merging of the droplets in the outlet tube (figure 6). Upon introducing an oil spacer at a flow rate of 200 *µ*l min^−1^, the inter-droplet spacing increases to approximately 500 *µ*m, effectively doubling the spacing from the case of no spacer, and substantially reduces the droplet merging in the outlet tube. Increasing the spacer flow to 400 *µ*l min^−1^ increases the spacing to nearly 800 *µ*m, at which point droplet merging at the outlet tube no longer occurs. A spacer flow rate of 600 *µ*l min^−1^ increases the inter-droplet spacing to approximately 2500 *µ*m in the channel, while a spacer flow rate above 600 *µ*l min^−1^ slightly disrupts droplet generation consistency, leading to non-uniform droplet generation at the inlet due to backflow.

### 3.4. Droplet detection and counting with computer vision

**Figure 7.**
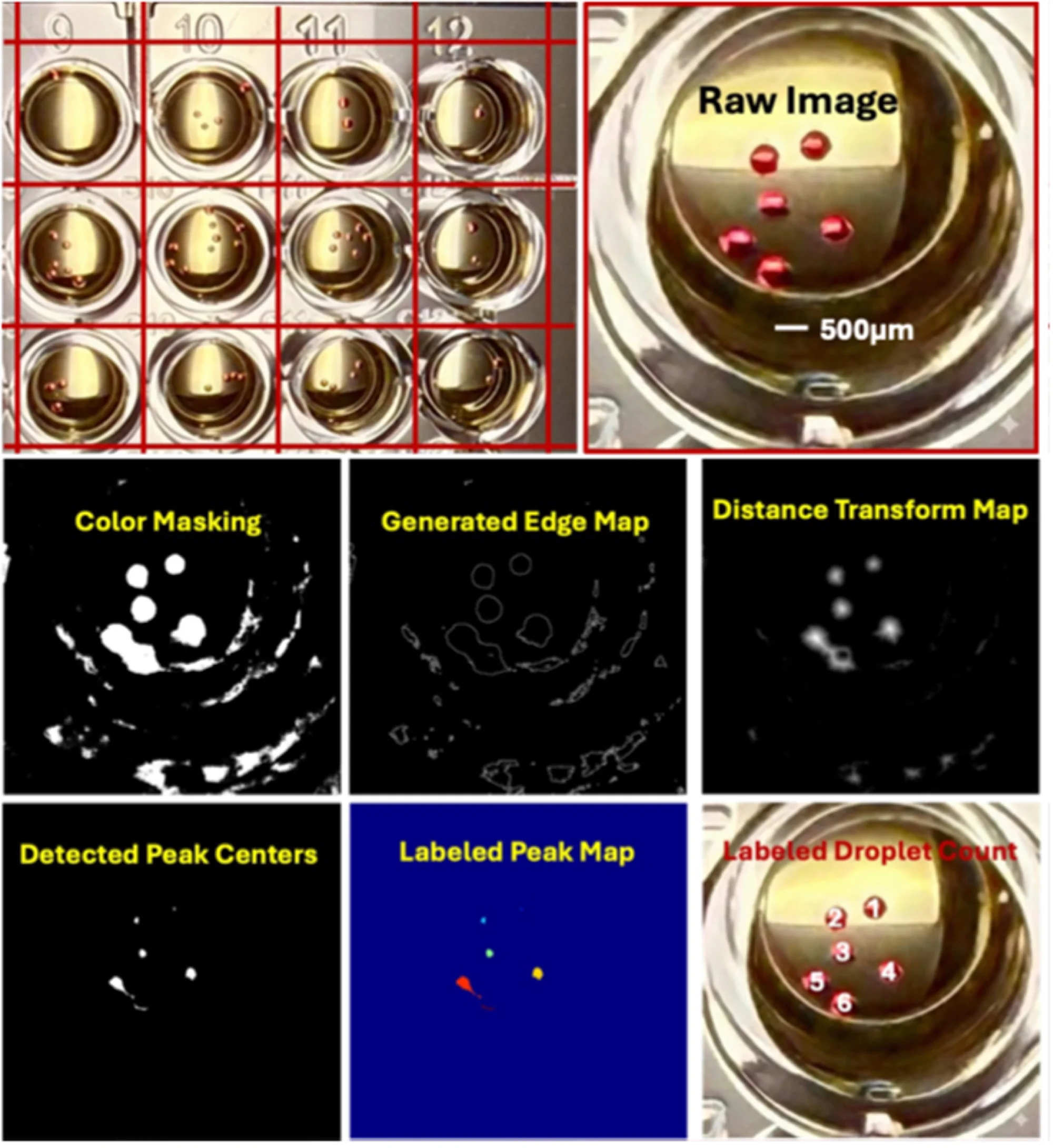
Automated computer vision pipeline for well-plate image processing and counting. (Top Left) Overview of a section of a microplate containing wells with varied numbers of red-dyed targets. A single well (Top Right), labeled ‘Raw Image’ and containing red spheres, is selected as the Region of Interest for the following analysis. Scale bar is 500 μm. (Middle) Successive image processing steps: ‘Color Masking’ creates a binary mask based on target color. The ‘Generated Edge Map’ uses Canny-like filters for boundary detection. ‘Distance Transform Map’ converts the edge map into a map of shortest distances from each point to the nearest edge, resulting in distinct intensity peaks for individual targets. (Bottom) Detection and labeling steps: ‘Detected Peak Centers’ identifies the locations of the targets based on the distance transform map. ‘Labeled Peak Map’ assigns unique object IDs to the identified targets using color-coding on a segmentation map. ‘Labeled Droplet Count’ provides the final output, showing original raw image with numbered target centers (1–6) for count verification. In this example, 6 targets are correctly identified. This pipeline enables rapid and high-fidelity volumetric analysis of samples without non-destructive interference.

The precision of downstream qPCR analysis is intrinsically linked to the volumetric consistency of the reagents partitioned into each well. Because single-cell gene expression profiling demands absolute quantification, determining the exact number of 100 nL droplets delivered per well is critical; any variance in droplet count directly alters the initial target cDNA concentration, which would propagate as artificial shifts in cycle threshold (${C_t}$) values. To achieve the statistical rigor required for clinical or GMP-level cell therapy validation, we developed an automated Python-based computer vision pipeline to verify these volumes *in situ* (figure 7). A comprehensive description of the underlying algorithmic layers, segmentation strategies, and image-processing configurations is detailed in the supplementary information.

Rather than relying on traditional colorimetric or fluorescent tracer dyes—which can inhibit polymerase activity or interfere with multiplexed optical channels—this image processing approach enables high-fidelity, non-destructive estimation of reagent volumes across the entire 96-well plate. Statistical analysis of the pipeline’s performance demonstrates exceptional partitioning fidelity, yielding an average of 4 droplets per well. This equates to a mean total reaction volume of 398 nL per well, achieving a final volumetric pass rate of 97.9% across the plate. Furthermore, the single-cell droplet manipulation architecture preserves a highly precise 1:9 mixing ratio of cDNA to master mix (10 nL:90 nl) with an observed accuracy of around 99.1%. Beyond minor stochastic exceptions, the system consistently delivers exactly four aqueous droplets per well, culminating in a highly uniform 400 nL total reaction volume. In the context of droplet microfluidic literature, high-throughput dispensing into conventional well plates frequently suffers from volume drifting or droplet splitting due to pinning effects at the nozzle tip. Achieving a discrete, uniform count of four droplets per well without satellite droplet generation represents a significant improvement in volumetric precision over manual low-volume pipetting and standard pneumatic dispensing arrays, ensuring highly reproducible mixing ratios for low-abundance transcripts.

### 3.5. Transcript detection following nanoliter microfluidic processing

To evaluate the integrity of the isolated cellular material and the effectiveness of the microfluidic-based electrocoalescence workflow, real-time qPCR was performed targeting both a housekeeping gene (*β*-actin) and RPE-associated markers including MITF1, MITF2, PMEL17, and PEDF. The qPCR assays were carried out on a QuantStudio™ 5 Real-Time PCR System using a commercially available SYBR Green master mix. The resulting *C*_t_ values confirmed successful amplification of the target transcripts after acoustic cell ejection, whole-transcriptome amplification, droplet generation, electrocoalescence, and collection into the 96-well plate format. Three independent qPCR plates were analyzed for each target gene, and the plate-level *C*_t_ values are summarized in table 1. Successful amplification was observed for the housekeeping gene *β*-actin as well as the RPE-specific markers MITF1, MITF2, PMEL17, and PEDF, confirming that the amplified cDNA remained suitable for downstream qPCR analysis following acoustic cell ejection, whole-transcriptome amplification, droplet generation, electrocoalescence, and automated collection. Because the objective of this study was to validate the feasibility of the proposed nanoliter microfluidic workflow rather than to perform quantitative comparison between independent samples, the *C*_t_ values are presented as representative plate-level results. Overall, these findings demonstrate that biologically relevant transcript signals can be successfully detected from nanoliter-scale reactions using the proposed microfluidic platform. The successful amplification and detection of *β*-actin together with robust expression of RPE-specific marker genes demonstrate that acoustic cell isolation and the subsequent microfluidic electrocoalescence workflow preserve cDNA integrity throughout sample processing.

**Table 1.** Plate-specific *C*_t_ values obtained using the microfluidic platform.

Target Gene	Plate-1 *C*_t_	Plate-2 *C*_t_	Plate-3 C_t_	Significance
β-actin	19.9	23.2	20.8	Housekeeping Control
MITF1	11.6	13.3	35.6	Developmental Identity
MITF2	22.5	21.0	28.9	Lineage Regulator
PMEL17	16.6	25.2	24.5	Pigmentation Matrix
PEDF	19.7	19.7	26.3	Secreted Growth Factor

These results validate the reliability of the nanoliter acoustofluidic–electrocoalescence platform for single-cell transcriptomic processing, confirming its capability to perform precise reagent mixing and downstream gene expression analysis without compromising molecular fidelity. Although digital droplet PCR (ddPCR) has been shown to provide superior sensitivity and absolute quantification for low-copy-number nucleic acid targets compared with conventional qPCR, the primary objective of this study was not to compare the analytical performance of these techniques. Instead, our goal was to demonstrate that the presented workflow enables reliable detection of extremely low quantities of nucleic acid using a widely available commercial qPCR platform (QuantStudio™ 5). By leveraging conventional qPCR instrumentation, the presented approach offers a practical and readily accessible solution that can be more easily adopted in routine laboratory and translational research settings.

## 4. Conclusions

The microfluidic platform described in this paper represents a transformative advancement in the characterization of RPE cell therapies. By successfully integrating nanoliter-scale droplet generation, DC electrocoalescence, and automated delivery into 96-well plates, the system addresses the critical limitations of conventional molecular assays. The platform’s ability to achieve 100% deterministic merging of 10 nL cDNA and 90 nL reagent master mixes ensure high sensitivity and reproducibility, which are essential for single-cell transcriptomic profiling. The successful amplification and detection of *β*-actin, along with the robust expression of RPE-specific marker genes, confirm that the integrated acoustic cell isolation and microfluidic electrocoalescence workflow maintains cDNA integrity throughout sample processing. Collectively, these findings establish the nanoliter-scale microfluidic platform as a reliable approach for single-cell transcript detection and molecular quality assessment of engineered RPE monolayers.

Furthermore, implementing a custom 2D gantry system and a Python-based computer vision model for volumetric verification provides the level of automation and statistical rigor necessary for integration into GMP workflows. The proposed workflow enables reliable detection of extremely low quantities of nucleic acid using a standard commercial qPCR platform, providing a fast, reliable, scalable, and readily accessible approach for single-cell molecular analysis. This manuscript demonstrates for the first time that droplet microfluidics offers an equally viable option for qPCR as traditional methods for RPE cells. The technology not only enhances the quality control resolution in RPE manufacturing but also provides a versatile tool for broader applications in single-cell biology and personalized medicine.

## Data Availability

The data cannot be made publicly available upon publication because no suitable repository exists for hosting data in this field of study. The data that support the findings of this study are available upon reasonable request from the authors. Supplementary data 1 available at: https://doi.org/10.1088/1361-6439/ae9c4b/data1.
